# Cryptorchidism in Boys With Cerebral Palsy Is Associated With the Severity of Disease and With Co-Occurrence of Other Congenital Anomalies

**DOI:** 10.3389/fendo.2018.00151

**Published:** 2018-04-16

**Authors:** Julia Spencer Barthold, Anton Wintner, Jennifer A. Hagerty, Kenneth J. Rogers, Md Jobayer Hossain

**Affiliations:** Nemours Biomedical Research/Division of Urology, Department of Orthopedics and Biostatistics Core, Nemours/Alfred I. duPont Hospital for Children, Wilmington, DE, United States

**Keywords:** cerebral palsy, cryptorchidism, congenital anomalies, orchidopexy, retractile testes

## Abstract

**Background:**

Cryptorchidism is reported in 40–50% of small case series of cerebral palsy (CP) and attributed to hypothalamic–pituitary–gonadal axis abnormalities, intellectual disability (ID), or cremaster spasticity. We collected demographic and clinical data to define the frequency of cryptorchidism and clinical comorbidities in a large CP population.

**Methods:**

Electronic health record data were collected for all male patients ≥7 years of age seen in a large, multidisciplinary CP clinic between 2000 and 2016. Variables including age, testicular position, surgical findings, CP severity, birth history, and comorbidities were tested for association using univariable and stepwise backward logistic regression analyses.

**Results:**

Of 839 established patients, testis position was scrotal in 553, undescended in 185 (24%), retractile in 38 (5%), and undocumented in 63 cases. Cryptorchidism were diagnosed at a mean age of 5.8 years, with 20% documented as acquired, and testes were most commonly in the superficial inguinal pouch (41%) and associated with an inguinal hernia (56%). Severity was bilateral in 114/166 (69%) undescended and 24/36 (66%) retractile cases, respectively. Mean birth weight and the frequency of prematurity (55, 58, and 54%) and multiple birth (14, 13, and 9%) were not significantly different among the three groups. We observed a strong ordinal trend in the frequency of comorbidities, including quadriplegia, syndromic features/known genetic disease, intrauterine growth restriction (IUGR), death, brain malformations, seizures, gastrostomy, absent continence, ID and hearing, speech or visual impairment, with the retractile group holding the intermediate position for the majority. The stepwise multivariable analysis showed independent positive associations of cryptorchidism with quadriplegia, syndromic features/known genetic disease, hearing loss, and absent continence, and inverse associations with gestational age and multiple birth.

**Conclusion:**

These data suggest that cryptorchidism is less common than previously reported in CP cases, but most strongly associated with quadriplegia. Delayed diagnosis may be related to an acquired condition or to the multiple additional functional deficits that occur in this population. Our data suggest that UDT and CP may both be components of malformation syndromes occurring in singleton births whose clinical features are more likely to include earlier delivery, IUGR, hearing loss, and/or global spasticity.

## Introduction

Cerebral palsy (CP) refers to a broad group of non-progressive motor disorders resulting from multifactorial, pre-, peri-, or postnatal central nervous system maldevelopment or injury. CP manifests as diplegic, hemiplegic, or quadriplegic spasticity, which is often associated with sensory deficits, intellectual disability (ID), and/or seizures (1, 2). CP is attributable to prenatal factors in the majority of cases (1). Risks include pre- or post-term birth, intrauterine growth restriction (IUGR), and twin gestation. Congenital anomalies (CAs) are present in 14–40% of CP cases, about five times higher than seen in the general population (1, 3, 4). Those affected do not have more severe forms of CP, but unlike individuals without CP, the risk of associated CAs is increased in children born full term. These may represent genetic syndromes, consistent with family studies supporting the heritability of CP (5, 6). While genetic association studies have failed to consistently implicate CP-specific loci (7), additional analyses identified potentially pathogenic rare exonic or copy number variants in 10–30% of patients (1, 8). Overall, the available data suggest that the etiology of CP is complex and heterogeneous.

In studies assessing the prevalence of CAs in association with CP, reproductive birth defects are reported rarely (3, 9) or not at all (10, 11). Underascertainment may be the result of underdiagnosis or undertreatment of a relatively minor anomaly in individuals with otherwise significant CAs, and physical or mental disabilities. Nevertheless, almost half of the recognized syndromes identified in CP patients in one series are known to include cryptorchidism as a component (9). Studies that have directly assessed the prevalence of cryptorchidism in males with CP with or without ID have focused on institutionalized individuals. Cryptorchidism was present in 36 of 88 (41%) and 21 of 39 (54%) of severely disabled males with CP in two small studies (12, 13). Similarly, the covariate adjusted multivariable risk ratio for CP was 20.9 in a perinatal cohort that included 385 cryptorchid boys (14). However, to date, no study of the prevalence of cryptorchidism in a large CP population has been reported. To provide potential insight into risk factors for cryptorchidism in subjects with CP, we took advantage of available electronic health record (EHR) data from a large, interdisciplinary clinic to more clearly define the prevalence of clinical characteristics in this subpopulation.

## Materials and Methods

We analyzed retrospectively retrieved EHR data for all male patients seen at the CP Center at the Alfred I. duPont Hospital for Children (AIDHC) from 2000 to 2016 following approval by the Nemours Office of Human Subjects Protection/Institutional Review Board with waiver of consent requirements. The earliest date coincided with initial EHR implementation at AIDHC. Pediatric subspecialists routinely see and fully examine CP clinic patients, and an extensive template was completed routinely for the majority of visits comprising information regarding full birth history and functional status for each patient. Additional data extracted from the EHR included other primary care visits at AIDHC-associated or other facilities, surgical histories and physicals, and scanned records from other institutions. Boys with a confirmed CP diagnosis who attended at least three clinic visits and were followed until at least age 7 (unless cryptorchidism was diagnosed before that time), were included.

Cerebral palsy cases were categorized as affected (with documentation of cryptorchidism and/or retractile testis at any time point) or unaffected. Subclassification in affected cases included testicular position, side (unilateral or bilateral), surgery (yes or no), and postoperative testicular position. Additional variables collected included race/ethnicity, gestational age, birth weight, birth history, CP severity; presence/absence of a movement disorder (athetoid or dystonic), postnatal injury or death, or at least one other CA (besides cryptorchidism) with or without a known genetic defect; and functional status related to vision and hearing (normal vs. abnormal), feeding (gastrostomy present vs. oral feeding), continence (present vs. absent), seizures (persistent or transient vs. none), speech (normal vs. abnormal or nonverbal), and cognition (characterized as normal/mild, moderate or severe/profound ID). We identified all examinations which documented testicular position, and recorded findings at the time of testicular surgery, when available. We summarized all study variables and analyzed their distribution in each of the case (undescended and retractile testes) and control (descended testes) groups. Categorical data were summarized using frequencies and percentages, and numerical data were summarized using means ± SDs. Two sample *t* tests and the Mann–Whitney *U* tests were used for parametric or non-parametric analysis of continuous variables, respectively, and Chi square tests were used to compare the distribution of categorical variables between affected and unaffected case groups.

We used a univariable logistic regression model to examine the potential association of study variables based on case classification. Odds ratios (ORs) with 95% confidence intervals (CIs) were reported for this purpose. We then used a stepwise backward method of multivariable logistic regression to select the variables significantly associated with the combined case group compared to the control group. All variables were included, and probabilities for entry and removal in this stepwise analysis were set to 0.05 and 0.10, respectively. Adjusted ORs with 95% CIs are reported for variables retained in each model. We used IBM SPSS version 22.0 for these statistical analyses, and all tests were two-tailed with the level of significance defined as <0.05.

## Results

Of 2,984 total records of boys identified through initial screening of the EHR data warehouse, 839 established male CP patients met our inclusion criteria. An additional 63 cases for whom available EHR data failed to provide documentation of testicular position during examination and/or of orchidopexy were excluded, leaving a total of 776 cases for analysis. Of these cases, 553 had descended, 185 (24%) had undescended, and 38 (5%) had retractile testes. The age in years at last follow-up was 13.9 ± 4.0 overall, and was slightly but significantly lower for boys with undescended testes (13.0 ± 4.3) as compared to boys with descended testes (14.3 ± 3.8) likely since follow-up to at least age 7 was not required for boys diagnosed with cryptorchidism at a younger age.

The average age at diagnosis of cryptorchidism was 5.8 ± 4.3 years, with 20% of cases documented as acquired, or descended on a prior examination. Retractile testes were first diagnosed at a mean age of 6.0 ± 3.6 years. The affected side was bilateral in 114/166 (69%) undescended and 24/36 (66%) of cryptorchid and retractile cases, respectively, for whom data were available. Of the 185 cryptorchid cases, 145 (78%) had surgery and operative reports were available for 97 (52%). Of these, 8 underwent a scrotal approach, and insufficient data documenting testicular position were available for another 79 cases. Documented testicular position was prescrotal or external ring (37%), superficial inguinal pouch (40%), inguinal or canalicular (15%), and abdominal (8%). An inguinal hernia was documented in 49 of 88 (56%) patients for whom information was available, of which 16 (33%) were repaired prior to the diagnosis of cryptorchidism. The majority (14, 88%) of boys who had an inguinal hernia repair that predated an ipsilateral orchidopexy were born preterm.

The frequency of prematurity (55, 58, and 54%) and multiple birth (14, 13, and 9%), and mean values for birth weight (2,280 ± 1,130, 2,023 ± 1,165, and 2,234 ± 1,141 g) and gestational age (34 ± 6, 33 ± 6, and 34 ± 6 weeks) were not significantly different among descended, retractile, and undescended testis cases, respectively. However, the prevalence of severe spasticity, ID, death, intrauterine growth restriction (IUGR), non-CNS CAs, brain malformations, requirement for gastrostomy, absent continence, seizures, hearing impairment, visual impairment, and abnormal speech were significantly different among the groups (Table 1). For the majority of variables, frequencies were intermediate for retractile as compared to descended and undescended testes and significance was enhanced for all variables when the descended and retractile testis groups were combined (Table 2). Of note, we identified 93 subjects (12%) who underwent placement of a baclofen pump for uncontrolled spasticity. The frequency of baclofen pump placement did not differ significantly between the undescended or retractile (14.3%) and descended (11%) testis groups (*p* = 0.2). Boys with baclofen pumps were more likely to have quadriplegia (87%; *p* < 0.001) and other severe CP phenotypes. We did not include this variable in multivariable models because of potential bias related to its elective nature.

**Table 1:** Frequencies (%) of clinical characteristics by cryptorchidism subtype in boys with cerebral palsy.

Variables (# cases)	Total	Descended	Retractile	UDT	*p*-Value**
Race (776)					
Caucasian	66.8	68.2	60.5	63.7	0.33
African-American	18.8	16.6	26.4	23.8	
Asian	1.8	2.0	2.6	1.1	
Other/unknown	12.6	13.2	10.5	11.4	
Diagnosis (776)					
Quadriplegic	42.3	32.5	47.4	70.3	<0.001
Hemiplegic	37.8	42.1	39.5	24.3	
Diplegic	20.0	25.3	13.2	5.4	
Movement disorder (776)	6.6	6.7	5.3	6.5	0.94
Death (776)	3.2	2.0	5.3	6.3	0.009
Gestational age <37 weeks (764)	54.3	55.4	57.9	53.6	0.85
Multiple birth (764)	12.5	14.0	13.2	8.7	0.18
Intrauterine growth restriction (IUGR) (728)	18.3	15.6	21.6	25.1	0.015
CA^a^ and/or genetic defect (764)	11.5	8.3	15.8	20.3	<0.001
Brain malformation (764)	9.7	8.1	10.5	14.3	0.049
Postnatal injury (766)	15.3	14.1	15.8	18.6	0.35
Hydrocephalus (776)	12.5	11.8	13.2	14.6	0.59
Gastrostomy (776)	29.3	21.7	31.6	51.4	<0.001
Absent continence (773)	41.9	33.0	43.2	68.5	<0.001
Seizures (776)	47.7	43.8	50.0	58.9	0.002
Hearing impairment (765)	12.0	8.0	7.9	25.4	<0.001
Visual impairment (771)	34.9	30.7	47.4	45.1	0.001
Abnormal speech (776)	47.8	41.2	50.0	67.0	<0.001
Intellectual disability (776)					
None-mild	43.7	51.5	36.8	21.6	
Moderate	31.6	29.8	31.6	36.8	
Severe/profound	24.7	18.6	31.6	41.6	<0.001

*^a^At least one non-CNS congenital anomaly (CA) in addition to cryptorchidism*.

**Results of chi square analysis.

**Table 2:** Frequencies (%) of subtypes and comorbidities in boys with cerebral palsy and descended or retractile vs. undescended testes.

Variables	Descended	UDT or retractile	*p*-Value**
Race			
Caucasian	68.2	63.2	0.1
African-American	16.6	37.0	
Asian	2.0	1.3	
Other/unknown	13.2	11.2	
Diagnosis			
Quadriplegic	32.5	66.4	<0.001
Hemiplegic	42.2	26.9	
Diplegic	25.3	6.7	
Movement disorder	6.7	6.3	0.83
Death	2.0	6.3	0.002
Gestational age <37 weeks	55.4	54.3	0.77
Multiple birth	14.0	9.5	0.09
Intrauterine growth restriction (IUGR)	15.6	24.5	0.004
CA^a^ and/or genetic defect	8.3	19.5	<0.001
Brain malformation	8.1	13.6	0.02
Postnatal injury	14.1	18.1	0.17
Hydrocephalus	11.8	14.3	0.32
Gastrostomy	21.7	48.0	<0.001
Absent continence	33.0	64.3	<0.001
Seizures	43.8	57.4	0.001
Hearing impairment	8.0	22.3	<0.001
Visual impairment	30.7	45.5	<0.001
Abnormal speech	41.2	64.1	<0.001
Intellectual disability			
Normal-mild	51.5	24.2	
Moderate	29.8	35.9	
Severe/profound	18.7	39.9	<0.001

*^a^At least one non-CNS congenital anomaly (CA) in addition to cryptorchidism*.

**p-Value: results of chi square analysis.

In univariable analyses (Table 3), estimated ORs for most variables were greater for undescended than for retractile cases. However, the differences were not significant except for quadriplegia, visual impairment, and severe/profound ID, most likely due to the small size of the retractile group. We found that the occurrence of cryptorchidism or retractile testis showed the strongest associations with quadriplegia, postnatal death, presence of at least one non-CNS CA with or without known genetic disease, absent continence, requirement for gastrostomy, hearing impairment, and severe/profound ID. All of these variables, except death, gastrostomy placement, and severe/profound ID, were retained in the multivariable analysis of undescended or retractile testes, which also included independent inverse associations with GA and multiple birth (Table 4). The results were similar for a model limited to undescended testes.

**Table 3:** Univariable logistic regression analysis comparing the prevalence of clinical characteristics/comorbidities based on cryptorchidism subtype.

	UDT or retractile		UDT		Retractile	
			
	OR	95% CI	OR	95% CI	OR	95% CI
Race						
Caucasian	Reference		Reference		Reference	
African-American	1.6	1.1, 2.3	1.5	1.0, 2.3	1.8	0.8, 3.9
Asian	0.7	0.2, 2.6	0.6	0.1, 2.6	1.5	0.2, 12.0
Other/unknown	0.9	0.6, 1.5	0.9	0.5, 1.6	0.9	0.3, 2.7
Diagnosis						
Diplegic	Reference		Reference		Reference	
Hemiplegic	2.4	1.3, 4.4	2.7	1.3, 5.5	1.8	0.6, 5.1
Quadriplegic	7.7	4.3, 13.6	10.1	5.1, 19.9	2.8	1.0, 7.7
Movement disorder	1.1	0.7, 1.8	1.1	0.7, 1.9	1.0	0.4, 3.1
Death	3.3	1.5, 7.4	3.4	1.5, 7.9	2.7	0.6, 12.8
Gestational age <37 weeks	0.96	0.7, 1.3	0.93	0.7, 1.3	1.1	0.6, 2.1
Multiple birth	0.6	0.4, 1.1	0.6	0.3, 1.0	0.9	0.3, 2.5
Intrauterine growth restriction (IUGR)	1.8	1.2, 2.6	1.9	1.1, 3.2	1.5	0.7, 3.4
CA^a^ and/or genetic defect	2.7	1.7, 4.2	2.8	1.8, 4.5	2.1	0.8, 5.2
Brain malformation	1.8	1.1, 2.9	1.9	1.1, 3.2	1.3	0.5, 3.9
Postnatal injury	1.3	0.9, 2.0	1.4	0.9, 2.2	1.1	0.5, 2.8
Hydrocephalus	1.3	0.8, 2.0	1.3	0.8, 2.1	1.1	0.4, 3.0
Gastrostomy	3.3	2.4, 4.6	3.8	2.7, 5.4	1.7	0.8, 3.4
Absent continence	3.6	2.6, 5.1	4.4	3.1, 6.3	1.5	0.8, 3.0
Seizures	1.7	1.3, 2.4	1.8	1.3, 2.6	1.3	0.7, 2.5
Hearing impairment	3.3	2.1, 5.2	3.9	2.5, 6.2	1.0	0.3, 3.3
Visual impairment	1.9	1.4, 2.6	1.8	1.3, 2.6	2.0	1.0, 3.9
Abnormal speech	2.5	1.8, 3.5	2.9	2.0, 4.1	1.4	0.7, 2.7
Intellectual disability						
None/mild defect	Reference		Reference		Reference	
Moderate	2.6	1.7, 3.8	2.9	1.9, 4.5	1.5	0.7, 3.3
Severe/profound	4.6	3.0, 6.8	5.3	3.4, 8.3	2.4	1.1, 5.3

*^a^At least one non-CNS congenital anomaly (CA) in addition to cryptorchidism*.

*OR, odds ratio; CI, confidence interval; descended is reference group in these analyses*.

**Table 4:** Stepwise logistic regression models to identify variables substantially associated with cryptorchidism subtype in boys with cerebral palsy.

Variable	Undescended or retractile testes		Undescended testes	
		
	Odds ratio (OR) (95% CI)	*p*-Value	OR (95% CI)	*p*-Value
Diagnosis				
Diplegia	Reference		Reference	
Hemiplegia	1.8 (0.95, 3.4)	0.07	2.0 (0.94, 4.2)	0.07
Quadriplegia	4.0 (2.0, 7.8)	<0.001	4.8 (2.2, 10.5)	<0.001
CA and/or genetic defect	2.5 (1.4, 4.3)	0.001	2.6 (1.5, 4.7)	0.001
Gestational age	0.96 (0.93, 0.99)	0.015	0.96 (0.92, 0.99)	0.023
Hearing impairment	1.9 (1.1, 3.1)	0.016	2.1 (1.3, 3.6)	0.005
Multiple birth	0.53 (0.29–0.96)	0.036	0.46 (0.23, 0.91)	0.025
Absent continence	1.6 (1.0, 2.6)	0.038	1.8 (1.1, 3.1)	0.021
Intrauterine growth restriction (IUGR)	1.5 (0.96, 2.3)	0.079	1.5 (0.94, 2.4)	0.085

*Variables included in the model: race, diagnosis, movement disorder, death, birth weight, gestational age, multiple birth, intrauterine growth restriction (IUGR), congenital anomaly (CA; non-CNS in addition to cryptorchidism) and/or genetic defect, postnatal injury, hydrocephalus, gastrostomy, absent continence, seizures, hearing impairment, visual impairment, abnormal speech, and intellectual disability*.

In view of these data, we explored the association of other CAs with cryptorchidism and CP in further analyses (Table 5). Of 144 CP cases with an associated brain malformation and/or at least one other non-CNS CA (18.8% of the total study group), 61 (42%) also had undescended or retractile testes (*p* < 0.001). We also found significant positive associations between CAs in the setting of CP with postnatal death, IUGR, term delivery, singleton birth, gastrostomy placement, absent continence, seizures, abnormal speech, and ID (Table 5). In a similar multivariable analysis of CAs in CP, we found independent positive associations with ID, undescended, or retractile testes, gestational age and IUGR, and a negative association with postnatal injury (Table 6).

**Table 5:** Frequencies (%) of subtypes and comorbidities in boys with cerebral palsy based on presence or absence of congenital anomalies (CNS and/or non-CNS) other than cryptorchidism.

Variables	Absent *n* = 620	Present *n* = 144 (18.8%)	*p*-Value*
Race			
Caucasian	67.1	68.1	0.97
African-American	19.0	19.4	
Asian	1.3	1.4	
Other/unknown	12.6	11.1	
Diagnosis			
Quadriplegic	41.5	47.2	0.26
Hemiplegic	37.7	37.5	
Diplegic	20.8	15.3	
Movement disorder	6.5	6.9	0.83
Died	2.6	6.3	0.026
Prematurity	58.8	36.4	<0.001
Multiple birth	13.9	6.3	0.013
Intrauterine growth restriction (IUGR)	16.3	27.0	0.003
Undescended or retractile testis	26.1	42.4	<0.001
Postnatal injury	16.5	10.4	0.07
Hydrocephalus	13.1	11.1	0.53
Gastrostomy	27.6	38.2	0.012
Absent continence	39.0	55.6	<0.001
Seizures	46.0	57.6	0.012
Hearing impairment	11.4	15.6	0.17
Visual impairment	34.2	39.9	0.20
Abnormal speech	44.8	61.8	<0.001
Intellectual disability			
None	47.1	27.1	<0.001
Moderate	30.0	38.9	
Severe/profound	22.9	34.0	

*p-Value: results of Chi square analysis.

**Table 6:** Stepwise logistic regression model of comorbidities associated with CNS and/or non-CNS congenital anomalies or genetic defect in boys with CP.

Variable	Odds ratio (95% CI)	*p*-Value
ID		
None/mild	Reference	
Moderate	2.1 (1.2, 3.4)	0.004
Severe/profound	1.9 (1.3, 3.1)	0.019
Undescended or retractile testis	2.0 (1.3, 3.1)	0.002
Gestational age	1.1 (1.1, 1.2)	<0.001
Postnatal injury	0.4 (0.2, 0.8)	0.006
Intrauterine growth restriction (IUGR)	1.5 (0.93, 2.4)	0.09

*Variables included in the stepwise logistic regression model: race, diagnosis, movement disorder, death, birth weight, gestational age, multiple birth, intrauterine growth restriction (IUGR), postnatal injury, hydrocephalus, gastrostomy, absent continence, seizures, hearing impairment, visual impairment, abnormal speech, intellectual disability (ID), and undescended or retractile testis*.

## Discussion

The present results suggest that common factors contribute to the risk of undescended or retractile testes in the CP population, and that the combined prevalence of 29% for males followed until at least age 7 is lower than identified in previous reports that focused solely on institutionalized, frequently older individuals. Our data also suggest that cryptorchidism is most strongly associated with spastic quadriplegia, with selected functional deficits including abnormal hearing and absent continence, and with non-CNS CAs in males with CP. Although we found associations between cryptorchidism and postnatal death, brain malformations, requirement for gastrostomy placement, seizures, visual impairment, abnormal speech and ID, none of these variables was retained in the multivariable model. This suggests that cryptorchidism in CP is more strongly associated with spasticity than with ID. This contrasts with the results of a prior population-based study showing independent association with both CP and ID, leading the authors to hypothesize that CNS lesions lead to hypothalamic–pituitary–gonadal axis dysfunction and failure of testicular descent (14). However, the total number of affected individuals in that study was small (18 with CP and 53 with ID) and the degree of overlap between the two diagnoses unclear. In the report by Cortada and Kousseff of 148 institutionalized individuals with ID, 44 (30%) had cryptorchidism and the majority of these (82%) also had CP (13). As in the present series, cryptorchidism was bilateral in the majority of cases. The similarity between the frequency distributions of the majority of variables that we analyzed and those reported previously in CP (1, 2, 4, 9–11, 15–22) supports the reliability of our data.

Smith and colleagues suggested a role for muscle spasticity in the etiology of cryptorchidism in boys with CP. In their prospective study, testicular position relative to the pubic tubercle of 50 boys with CP (25 aged <2.5 and 25 aged 5–10 years) was higher than that of aged-matched controls, although no cryptorchidism was reported (23). The authors theorized that progressive cremaster muscle shortening may, therefore, account for cases of acquired cryptorchidism in the CP population. Our documentation of a prior scrotal position for 20% of subsequently cryptorchid testes and the average age at diagnosis (5.8 years) suggest that testicular ascent may be common in the CP population. Failed or delayed diagnosis could also be related to deferral of testicular exams in patients with multiple medical concerns, or potential uncertainty regarding the need for treatment in this population (24, 25). We also observed apparent acquired cryptorchidism in boys who had undergone prior inguinal hernia repair, but biopsy data in this population has suggested that maldescent in these cases is likely primary and not truly iatrogenic (26). In a comparison of the present data and those for acquired cryptorchidism, we found that both sides are more commonly affected (69%) in CP, but the milder phenotype (77% of testes distal to the external ring) and the prevalence of associated inguinal hernia (56%) are similar to those we reported for uncomplicated cases of testicular ascent (79 and 46%, respectively) (27). It remains unclear if cremaster hyperactivity in otherwise normal children and spasticity in boys with CP both predispose to testicular ascent.

The present study, although retrospective, comprises the largest study of testicular position in a CP population to date, providing increased power to detect factors independently associated with cryptorchidism through multivariable statistical analysis. Our results suggest that cryptorchidism is more likely to occur in cases of CP associated with other CAs and with IUGR, a known strong risk factor for both non-syndromic cryptorchidism (28, 29) and for CP (30, 31). Reciprocally, we also identified cryptorchidism and IUGR as independent variables associated with the occurrence of other CAs in cases of CP. These shared associations are consistent with increasing evidence that CAs and genetic defects are both more common than previously recognized in boys with CP (1, 8, 22). Nevertheless, we did observe differences in the patterns of association of CP with cryptorchidism and with other CAs. While both presentations were more common in singletons, that association was not retained in the final model for CAs (Table 6). Cryptorchidism risk in both this study and the general non-CP population (28, 29) is associated with decreased gestational age. In contrast, we found an increased frequency of other CAs in children with CP born near term, as reported previously (1, 11). Similarly, unlike cryptorchidism, we found that the occurrence of other CAs did not correlate with the pattern of spasticity, a finding noted by others who studied the association of non-CNS CAs with CP (11).

Other independent associations that we observed for cryptorchidism in CP include hearing deficit and absence of continence. The reported prevalence of hearing loss in a large cohort of children with CP (*n* = 685) is the same as we found in our population (12%), and its occurrence was associated with quadriplegia, ID, movement disorders, visual impairment, and seizures in univariable analyses (20). However, the causes, which may be genetic or environmental, remain largely unknown. Similarly, a study of children with CP (*n* = 79) and varying degrees of urinary incontinence suggested association with ID, severity of spasticity, movement disorder, and issues related to speech and swallowing in univariable analyses, and with ID and more profound functional impairment in a multivariable model (32).

In conclusion, our data suggest that the prevalence of cryptorchidism in males with CP is approximately 10-fold greater than that of the general population, but lower than previous estimates. We found that its occurrence is most commonly bilateral and associated with measures of increased severity and with the co-occurrence of other CAs. Additional factors that may contribute to a higher risk of cryptorchidism in CP include cremaster spasticity, prematurity, and possibly inguinal hernia repair in infancy. Although testes often descend spontaneously in otherwise normal premature boys, it is possible that comorbidities reduce the likelihood of postnatal descent in boys with CP. Diagnosis in later childhood due to apparent testicular ascent is possible; therefore, serial examination is indicated. The combination of cryptorchidism, other CAs and IUGR may indicate a higher risk of genetic causation in individuals with CP, but further studies are needed. However, the available evidence suggests that the etiologies of both cryptorchidism and CP are complex and multifactorial.

## Ethics Statement

This study was approved by the Institutional Review Board (IRB #262462) of Nemours and requirement for informed consent/parental permission and assent, and authorization for use and disclosure of protected health information was waived based on the retrospective nature of the approved research.

## Author Contributions

JB designed the study, collected and analyzed data, and wrote the manuscript; AW and KR collected data and approved the manuscript; JH edited and approved the manuscript; MH contributed to study design, analyzed data, edited, and approved the manuscript.

## Conflict of Interest Statement

The authors declare that the research was conducted in the absence of any commercial or financial relationships that could be construed as a potential conflict of interest.
